# Technological innovations and high-throughput applications of light-sheet microscopy

**DOI:** 10.52601/bpr.2025.250013

**Published:** 2026-02-28

**Authors:** Jie Wang, Yan chen Liu, Peng Fei

**Affiliations:** 1 School of Optical and Electronic Information, Wuhan National Laboratory for Optoelectronics, Huazhong University of Science and Technology, Wuhan 430074, China

**Keywords:** LSFM, High-throughput, Sample compatibility, Hyperspectral

## Abstract

Light-sheet fluorescence microscopy (LSFM), with its innovative design of selective plane illumination and orthogonal detection optics, significantly reduces phototoxicity and photobleaching inherent in conventional microscopy, providing a revolutionary tool for long-term dynamic imaging of living specimens. This review focuses on throughput enhancement strategies of LSFM, systematically summarizing advancements in optical architecture optimization and multimodal integration. Key technological innovations include: improved sample compatibility, large-field imaging via optimized light-sheet generation, microfluidics-coupled high-throughput automation, and hyperspectral imaging for multiplexed analysis. Through adaptive light-sheet modulation, remote focusing synchronization, and AI-driven algorithmic optimization, LSFM achieves multiscale 3D imaging spanning subcellular structures to centimeter-scale tissues at speeds exceeding hundreds of volumetric frames per second. In biomedical applications, LSFM has successfully resolved complex processes such as cellular lineage dynamics during embryogenesis, whole-brain neuronal activity mapping, and structure-function correlations in cardiovascular systems, while enabling high-throughput drug screening and pathological model analysis. These breakthroughs establish LSFM as a cornerstone technology for intravital imaging, offering an integrated solution that combines high spatiotemporal resolution, minimal photodamage, and big-data throughput. By bridging molecular, cellular, and organ-level observations, LSFM drives paradigm shifts in developmental biology, neuroscience, and translational medicine, empowering unprecedented exploration of living systems across scales.

## INTRODUCTION

Fluorescence microscopy techniques, including widefield and confocal microscopy, have been essential tools in life science research, enabling the analysis of cellular and tissue structures and functions. However, traditional epifluorescence illuminates the entire three-dimensional volume of a sample, leading to significant out-of-focus signal, phototoxicity, and photobleaching, which severely limit the ability to perform high-resolution, long-term dynamic imaging of live samples.

Light-sheet fluorescence microscopy (LSFM) addresses these challenges through an innovative optical design. Instead of full-volume illumination, LSFM generates an ultrathin light sheet that selectively excites fluorescence in a single plane of the specimen. The detection system then collects emitted light orthogonal to the illumination plane. This approach reduces out-of-focus signals while delivering three to five orders of magnitude less light energy than confocal microscopy, thereby suppressing both photobleaching and mitochondrial phototoxicity, establishing LSFM as the optimal modality for live-cell imaging.

First demonstrated as a diffraction-limited technique in 2004 (Huisken *et al.* 2004), LSFM has rapidly evolved over two decades into a powerful imaging modality. By 2014, its transformative potential earned recognition as “*Method of the Year*” by *Nature Methods*, which compiled key studies and highlighted its transformative impact on biological imaging (Stelzer 2015). This recognition marked LSFM’s pivotal role in enabling high-speed, low-phototoxicity, and high-throughput imaging of live cells and large-volume samples.

The hallmark features of LSFM – high imaging speed and minimal phototoxicity – render it exceptionally suited for 3D imaging of biological specimens, especially in long-term dynamic observations of live cells and large samples (Panier *et al.* 2013). In cell biology, LSFM has proven instrumental in investigating organelle reorganization under stressors like drug exposure and pathogen infection, while capturing dynamic processes including cell migration, division, and differentiation (Huisken *et al*. 2004; Huisken and Stainier 2009). Crucially, its high-throughput capacity facilitates the rapid acquisition of large-scale three-dimensional data, creating new paradigms for quantitative analysis of cellular phenotypes and biological networks.

Building upon these inherent advantages and sustained technical innovations, LSFM has demonstrated transformative potential across diverse domains ranging from embryogenesis to immune surveillance and pathophysiological modeling. This review focuses specifically on throughput enhancement strategies in light-sheet microscopy, covering both optical system optimizations and spectral dimension expansions.

## USABILITY DEVELOPMENTS IN LIGHT-SHEET MICROSCOPY

Conventional imaging platforms (Chhetri *et al.* 2015; Huisken *et al.* 2004; Huisken and Stainier 2009; Keller *et al.* 2008; Keller and Ahrens 2015; Panier *et al.* 2013; Truong *et al.* 2011) impose inherent constraints on sample mounting approaches, primarily dictated by their optical architecture and working distance limitations. This inherent incompatibility precludes the direct application of standard laboratory consumables (*e*.*g*., multi-well plates, glass slides), mandating protocol modifications that degrade both system compatibility and imaging throughput. Dual-objective configurations typically demand specialized sample preparation to meet optical alignment requirements, creating compatibility barriers that reduce throughput capacity. LSFM, however, demands flexible and efficient sample handling to fully utilize its high-speed and high-resolution capabilities. The open-top architecture overcomes these limitations by removing lateral spatial constraints, enabling imaging of unconventional specimens incompatible with traditional systems. This design paradigm enhances throughput and operational flexibility through native compatibility with diverse mounting configurations. Additionally, single-objective systems provide enhanced compatibility with commercial microscope platforms, standardizing workflow efficiency.

The evolution of light-sheet microscopy architectures demonstrates concerted engineering efforts to address these fundamental constraints. To bridge this gap between optical performance and practical utility, Wu *et al*. conceived inverted selective plane illumination microscopy (iSPIM), ingeniously merging light-sheet excitation with inverted microscope geometry (Wu *et al.* 2011). This configuration established compatibility with conventional flat sample formats (*e*.*g*., coverslips, multiwell plates), streamlining sample handling while enhancing throughput. The iSPIM platform achieved unprecedented volumetric imaging rates of 0.5 Hz (1 vol/s) with a volume size of 360 × 360 × 40 pixels (57.6 × 57.6 × 40 μm^3^), representing a 30-fold improvement over spinning-disk confocal microscopy in temporal resolution. This speed enabled continuous imaging of *Caenorhabditis elegans* embryos over 14 h without detectable phototoxicity.

Building on this, Wu *et al*. engineered dual-view selective plane illumination microscopy (diSPIM) (Wu *et al.* 2013b), which employed two orthogonal objectives, achieving 330 nm isotropic resolution at 200 fps – a critical advance enabling quantitative analysis of subcellular motility events. This advancement allowed researchers to observe fast cellular dynamics, such as axon growth and cell migration, at unprecedented temporal and spatial resolutions. The reflective diSPIM variant (Wu *et al.* 2017b) incorporated dielectric-coated coverslips to boost imaging speed to 400 fps (2× enhancement) while acquiring four complementary views, improving temporal resolution and collection efficiency. This architecture enhanced both temporal resolution and light collection efficiency while optimizing isotropy, ultimately delivering 260 nm lateral and 300 nm axial resolution. These developments established a robust framework for high-resolution volumetric imaging of calcium signaling events and organelle interactions across varied biological specimens.

To overcome spatial constraints and enhance adaptability, McGorty *et al*. pioneered the open-top selective plane illumination microscope (open-top SPIM) (McGorty *et al.* 2015). This design integrated aberration-correcting water prisms with inverted optical paths, maintaining 45° coverslip geometry while optimizing both operational flexibility and acquisition throughput. The system achieved high-throughput 3D imaging of 32 *Drosophila* embryos within 2.3 min, while sustaining developmental observation over 12 h with minimal photodamage.

Building on this foundation, the multi-immersion open-top light-sheet microscope (OTLS) introduced in 2019 further advanced the field (Glaser *et al.* 2019). This system resolved chronic immersion medium compatibility constraints through its scalable planar sample stage with a modular architecture, enabling macroscopic imaging domains. This design enabled mounting samples of diverse sizes and shapes without complete immersion, reducing contamination risks and streamlining workflows. OTLS supported multiple tissue-clearing protocols and refractive index matching strategies, significantly improving compatibility with a wide variety of specimens. The platform attained volumetric imaging rates of 1 mm^3^/min across unprecedented spatial domains (10 × 10 × 0.5 cm^3^), surpassing the capabilities of open-top SPIM in both sample capacity and volumetric imaging speed.

In high-magnification regimes constrained by working distance requirements, single-objective systems have revolutionized optical sectioning approaches. Dunsby introduced Oblique Plane Microscopy (OPM) (Dunsby 2008), a novel optical sectioning technique that combines tilted selective plane illumination with oblique imaging. Utilizing a single high-numerical-aperture objective lens to simultaneously illuminate and image the sample, OPM obviated the need for a separate illumination lens in the sample plane. This optical arrangement generated tilted illumination planes through controlled angular modulation of the excitation beam, effectively decoupling illumination geometry from detection optics while streamlining system complexity.

Reflective light-sheet microscopy uses 45° micromirrors to achieve illumination perpendicular to the optical axis, eliminating the need for remote focusing systems. In 2013, Gebhardt *et al*. utilized atomic force microscope (AFM) cantilever reflection to develop a reflective light-sheet system, where obliquely incident light sheets were reflected by the cantilever to illuminate the sample vertically (Gebhardt *et al.* 2013). In 2018, Ponjavic *et al*. further optimized the design, constructing a compact single-objective system using AFM cantilevers as reflective interfaces that avoided refocusing when moving the sample stage (Ponjavic *et al.* 2018). Li *et al*. combined such reflective systems with nanopipettes to achieve synchronized single-molecule delivery and imaging. This technology is compatible with high numerical aperture objectives and can directly image planar samples like multi-well plates, serving as an important complement to tilted plane systems (Li *et al.* 2021).

In modular integration solutions, the soSPIM system developed by Remi Galland's team in 2015 achieved single-objective light-sheet imaging on standard inverted microscopes by integrating micromirror chambers with laser beam steering units (Galland *et al.* 2015). Its core technologies included: replaceable micromirror chips adaptable to various sample sizes, dynamic adjustment of light-sheet thickness/size, and 3D super-resolution imaging driven by single-molecule detection (with localization accuracy reaching mainstream levels). The system supported dual-color embryo imaging and live cell dynamic observation, and its modular design allowed rapid switching between imaging modes without recalibration.

Pioneered by Hillman's group in 2015, SCAPE microscopy (swept confocally aligned planar excitation) redefined single-objective imaging paradigms through its integrated illumination-detection architecture (Bouchard *et al.* 2015). SCAPE employs a single high-numerical-aperture lens to simultaneously generate the light sheet and image the sample. By incorporating a scan-descan configuration, SCAPE achieved three-dimensional volumetric imaging without requiring sample translation. Subsequent refinement through SCAPE 2.0 (Voleti *et al.* 2019) achieved volumetric rates exceeding 300 vol/s, demonstrating whole-organ imaging capacity in cleared specimens including intact murine brains.

Kumar *et al*. conceived the epi-illumination SPIM (eSPIM) (Yang *et al.* 2019) architecture, featuring an innovative fusion of light-sheet excitation and epifluorescence collection within a single high-NA water-immersion objective. Combined with an optimized remote imaging module, eSPIM overcame the numerical aperture loss seen in traditional LSFM systems, attaining 316 nm lateral and 443 nm axial resolution under Bessel beam illumination. The system demonstrated native multiwell plate compatibility, supporting the parallel acquisition of 16 wells at 14.7 vol/s, with continuous dynamic observations lasting up to 8 h.

Ke Xu’s team developed the obSTORM system to address the imaging depth limitations of single-molecule localization microscopy (SMLM) (Kim *et al.* 2019). By optimizing PSF ellipticity through water immersion objectives and polarization beam splitters, combined with tilted light-sheet illumination and OPM architecture, they achieved 44 nm resolution imaging of A549 cell microtubules at depths up to 66 μm, and successfully resolved thick tissue samples like *C*. *elegans* gonads and *Drosophila* brains.

In 2020, Sapoznik *et al*. substituted the water-immersion objective with a custom glass-tipped tertiary objective (Sapoznik *et al.* 2020), enabling volumetric acquisition at 800 optical planes per second. Building on this foundation, Yang *et al*. engineered the DaXi platform with dynamic stage-light sheet synchronization (LS^3^ technology) for active plane stabilization (Yang *et al.* 2022). This system achieved a 3000 × 800 × 300 μm^3^ imaging volume, simultaneously capturing nine zebrafish embryos with subcellular resolution (lateral: 0.7 μm; axial: 1.5 μm) at 5-Hz temporal sampling.

The latest advancement is the high-throughput Bessel oblique plane microscopy (HBOPM) system, introduced by Wang *et al*. (Wang *et al.* 2024). This novel system combines advanced microfluidic chips and automated intelligent imaging algorithms, achieving significant improvements in long-term 3D dynamic imaging and throughput. By integrating a microfluidic chip with automated intelligent imaging algorithms, HBOPM achieved long-term 3D dynamic imaging of over 400 CAR-T cell and tumor cell pairs for up to 5 h. This system, compatible with the commercial IX83 inverted microscope, captured key parameters such as immune synapse formation, microtubule polarization, and actin dynamics at speeds of 2.5 vol/s. HBOPM's automated data analysis supported high-throughput extraction of cellular phenotypes, overcoming the low-throughput limitations of conventional LSFM.

Additionally, in 2025, Amine Driouchi *et al*. used a robust single-objective illumination and detection mode – Oblique Line Scanning (OLS), achieving nanoscale spatial resolution and sub-millisecond temporal resolution across large fields of view (Driouchi *et al.* 2025). They successfully captured protein movement up to 14 μm^2^/s in live cells. These technologies, through optical path reconstruction, reflective interface innovations, and hardware modularization, are gradually breaking through the physical limitations of single-objective systems (Cheng *et al.* 2024), providing multidimensional tools ranging from subcellular single-molecule localization to organ-scale imaging.

In 2024, Yannan Chen *et al*. developed a low-cost, scalable, and versatile light-sheet fluorescence microscopy framework called “projection light-sheet microscopy (pLSM)” (Chen *et al.* 2024). They constructed the pLSM system using consumer-grade components, an optimized optical system, a web-based control architecture, and software-driven light-sheet modulation technology. This system enables high-quality imaging of diverse samples, including mouse brains, human brain specimens, organoids, and bacterial biofilms processed with different methods.

**Figure 1:**
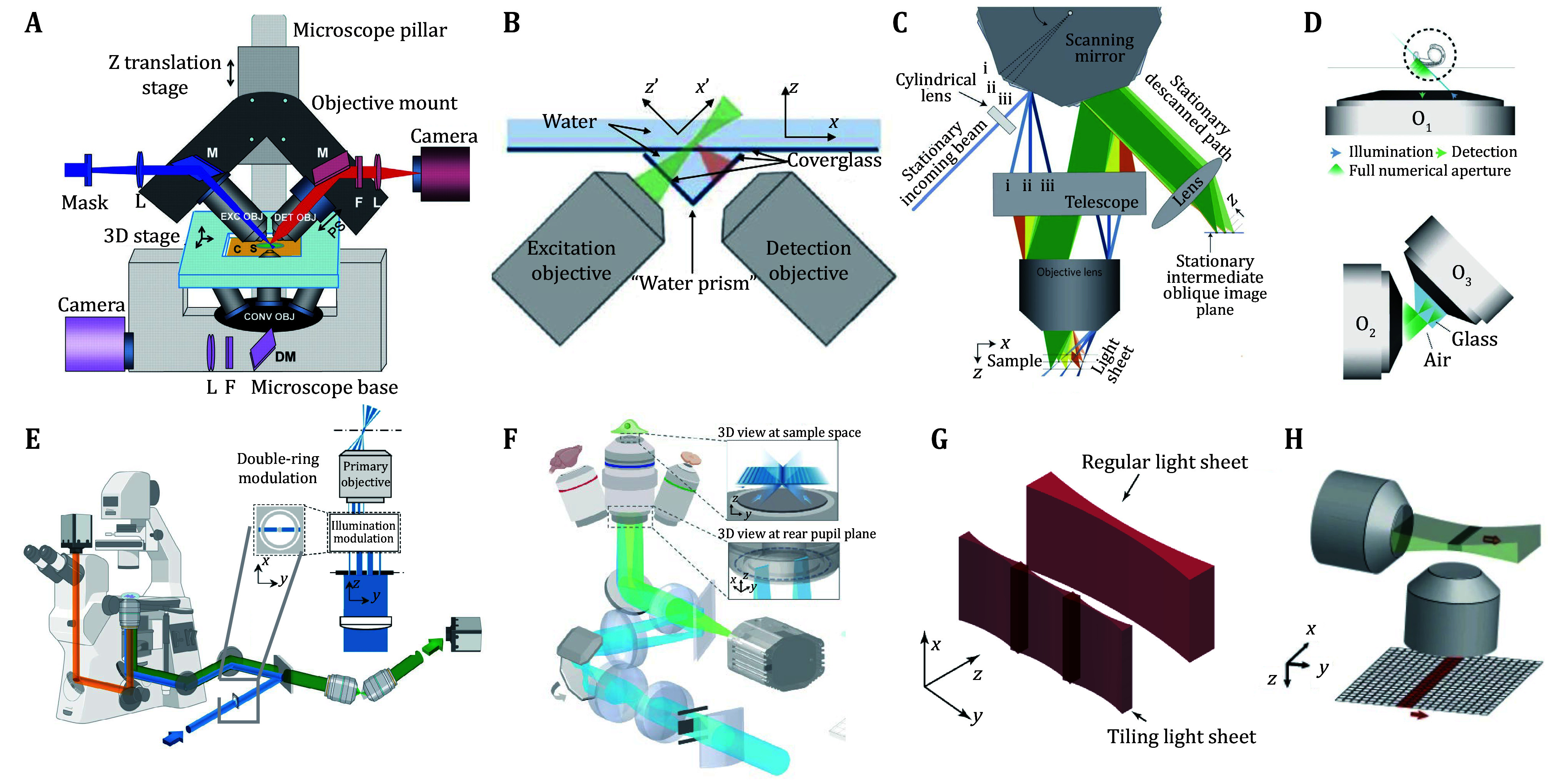
The modality of light sheet microscope. **A** iSPIM (Wu *et al.* 2011). **B** Open-top SPIM (McGorty *et al.* 2015). **C** SCAPE (Bouchard *et al.* 2015). **D** DaXi (Yang *et al.* 2022). **E** HBOPM (Wang *et al.* 2024). **F** TIM (Fei *et al.* 2024). **G** TLS-SPIM (Fu *et al.* 2016). **H** ctASLM (Voigt *et al.* 2019)

The scalability of pLSM is reflected in its web-based imaging workflow, which allows remote control of multiple microscopes. Its cost advantage is achieved through the use of affordable components such as pocket laser projectors and Nvidia Jetson Nano developer boards, significantly reducing system cost and complexity. This technology provides a more accessible, economical, and scalable high-resolution imaging solution for biomedical research (Table 1, Fig. 1).

**Table 1:** Summary of the comparison of characteristics and applications of light sheet microscopy techniques in the biomedical field

Technical name	Core parameters	Advantages	Limitations	Applicable biomedical scenarios
SPIM	Resolution: 1.1 μm; Field of view: mm; Imaging speed:1−4 planes/s	Low noise; Low photodamage	Limited sample preparation	Cardiac function research
OPM	Resolution: 0.67 μm	Easy to operate	Relatively reduced numerical aperture	Microfluidic technology research
SCAPE 2.0	Resolution: 0.75 × 0.24 × 0.19 μm³ (*x*, *y*, *z*); Imaging speed:300 vol/s	Fast imaging speed; Single-objective design, convenient for operation	Complex system configuration	Neuroscience research
diSPIM	Resolution: 330 nm; Imaging speed: 200 planes/s	Suitable for observing fast dynamic processes	Complex device, high cost	Cell biology research
DaXi	Lateral resolution: 450 nm; Field of view: 3000 × 800 × 300 μm^3^	Multi-view imaging function	May not fully utilize its large field of view for very small samples	Developmental biology research
eSPIM	Resolution: 339 ± 18 nm; Field of view: 100 × 70 × 20 μm^3^; Imaging speed: 14.7 vol/s	Compatible with common biological sample holders, convenient for high-throughput experiments	Complex optical design and adjustment of the system, requires professional technology	Cell biology research
OTLS	Resolution: 700 nm; Imaging speed:1 mm³/min	Easy to operate and integrate	Relatively small imaging volume	Cell biology research
Open-top SPIM	Resolution: 1.1 ± 0.03 μm	Capable of imaging multiple samples	Poor imaging effect for highly scattering samples	Embryo development research
HBOPM	Resolution: 320 nm; Imaging speed: 2.5 vol/s	Capable of observing a large number of cells simultaneously	Complex structure	Cancer immunotherapy research

## INTEGRATION OF LIGHT-SHEET MICROSCOPY WITH OTHER SYSTEMS

Open-top and single-objective configurations now interface natively with throughput-enhancing platforms including SPIM-fluid hydrodynamic focusing, flow cytometric excitation synchronization, and microfluidic multiplexing. Such hybrid architectures enable accelerated acquisition rates while preserving subcellular resolution – a prerequisite for high-content drug screening campaigns. By automating specimen delivery and data acquisition, these systems reduce hands-on preparation from minutes to seconds, establishing LSFM as a cornerstone technology for multiscale biological interrogation spanning organoids to population-level single-cell analyses.

Building on earlier flow-cytometric principles, Wu *et al*. engineered a light-sheet-based three-dimensional flow cytometer (Wu *et al.* 2013a), which guided samples through the light sheet plane to enable rapid 3D imaging without the need for sample fixation, offering an efficient solution for the rapid analysis of large-scale samples. McGorty *et al*. pioneered the SPIM-fluid platform, employing fluorinated ethylene propylene (FEP) tubing for hydrodynamic sample delivery (Gualda *et al.* 2015). This innovation abolished agarose embedding, slashing preparation time from over 15 min per sample to just a few seconds. Additionally, the platform integrated automated multi-well plate loading systems and motorized syringes, greatly increasing sample throughput and enabling the simultaneous processing of thousands of samples. This design made SPIM-fluid particularly suitable for high-throughput applications, such as drug screening and large-scale population studies.

The field advanced with Paiè *et al*.’s light-sheet microfluidic chip, which exploited hydrodynamic focusing to bypass mechanical scanning (Paiè *et al.* 2016). By integrating cylindrical lenses and flow-aligned illumination, this system achieved 30 samples/min imaging at cellular resolution. In comparison, Sala *et al*. developed a “microscope-on-a-chip” system (Sala *et al.* 2020) that integrated light-sheet microscopy with microfluidics on a single chip, enabling automated specimen transport and enhanced illumination homogeneity. This innovation streamlined workflows through lower-cost fabrication and native compatibility with commercial inverted microscopes, establishing it as a scalable platform for single-cell interrogation.

## HIGH-THROUGHPUT ILLUMINATION METHODS IN LIGHT-SHEET MICROSCOPY

Light-sheet modulation enhances throughput by enabling a larger field of view (FOV), particularly crucial for centimeter-scale tissue imaging. An optimized light sheet maximizes lateral coverage (*x*-*y* plane) while minimizing axial thickness (*z*-axis). The FOV along *x* (FOV_*x*_ = 2Z_R_) is governed by Rayleigh length, while FOV_*y*_ is constrained by illumination optics. The *x*-axis field of view is conventionally defined as twice the Rayleigh length. This dual-parameter framework defines the system's maximum FOV. Increasing the Rayleigh length results in a decrease in axial resolution, so there is a trade-off between the two.

In 2015, Gao engineered a light-sheet SPIM system employing light-sheet tiling to boost throughput for large-scale samples (Gao 2015). This design expanded the FOV by dynamically translating light sheets across the imaging plane. However, the static light-sheet configuration during imaging prevented dynamic parameter adaptation, constraining its performance on heterogeneous or structurally diverse samples.

Building on Gao’s static light-sheet approach, Fu *et al*. addressed the critical need for real-time adaptability in SPIM systems. In 2016, Fu *et al*. introduced a tiling light-sheet SPIM (TLS-SPIM) (Fu *et al.* 2016) with dynamic parameter optimization, enabling real-time light-sheet adjustment based on feedback. This innovation enhanced illumination uniformity and sample adaptability – critical for imaging complex specimens such as zebrafish and *C. elegans* embryos. By integrating multiple light-sheet modes (Gaussian, Bessel, lattice), TLS-SPIM achieved superior sample compatibility and imaging flexibility compared to Gao’s system.

In 2020, Chen *et al*. engineered a light-sheet microscope specifically for large cleared tissues (Chen *et al.* 2020), achieving an unprecedented resolution range from microns to sub-100 nanometers. This platform enabled multicolor 3D imaging with semi-automated operation, compatibility across tissue-clearing methods, and adaptive optics that reduced background noise. Compared to Fu’s TLS-SPIM, which was optimized for millimeter-scale samples, Chen’s platform represented a paradigm shift in large-tissue imaging of centimeter-scale samples, delivering superior adaptability and throughput.

Compared to the tiling light-sheet microscopy technology, which primarily focuses on expanding the FOV for high-throughput imaging of complex samples, mesoSPIM and ctASLM systems achieve significant improvements in axial resolution, optimizing imaging performance for large-scale optically cleared samples.

In 2019, Voigt *et al*. engineered mesoSPIM – an open-source light-sheet microscopy platform (Voigt *et al.* 2019). By integrating high-speed Gaussian light sheets with axial scanned light-sheet microscopy (ASLM), the system achieved uniform illumination while eliminating shadow artifacts. Optimized for centimeter-scale specimens (*e*.*g*., whole mouse brains), mesoSPIM delivers whole-organ 3D imaging at 6.5 μm resolution within 8 min. In the same year, Chakraborty *et al*. engineered cleared-tissue axially swept light-sheet microscopy (ctASLM) (Voigt *et al.* 2019), a breakthrough platform integrating adaptive optics and axial scanning to overcome scattering artifacts in cleared specimens. ctASLM achieves 260 nm isotropic resolution across millimeter-scale fields of view, enabling volumetric reconstruction of subcellular architecture in chemically cleared tissues, from neuronal synapses in mouse brains to organelle distribution in human organoids. Together, mesoSPIM and ctASLM synergistically enhance volumetric throughput, spanning rapid centimeter-scale mapping and subcellular-resolution imaging, thereby enabling high-throughput multiscale analysis of cleared tissues in large biological systems.

## ABERRATION CORRECTION IN LIGHT SHEET MICROSCOPY

Spherical aberration arises from the inconsistent focusing of marginal and central rays due to the spherical shape of lenses during light-sheet propagation. In large FOV scenarios, uneven light-sheet thickness exacerbates this issue, degrading axial resolution and imaging uniformity. In traditional selective plane illumination microscopy, static light sheets generated by cylindrical lenses often accumulate spherical aberration due to edge divergence. Replacing static with dynamic light sheets via digitally scanned laser light microscopy (DSLM), which reduces edge divergence through high-speed scanning, improves light-sheet uniformity and mitigates spherical aberration. For large FOV applications like axial scanning light-sheet microscopy (ASLM), Kebin Shi *et al*. introduced aberration-free tunable foci technology (ATF-GS) based on the Gerchberg-Saxton algorithm, constructing a closed-loop phase modulation system via numerical light modulation (Wang *et al.* 2025). By optimizing for target axial intensity distributions and expanding the computational plane to XZ/YZ dimensions, the algorithm iteratively refines incident wavefront phases through feedback, resolving multifocal artifacts and spherical aberration caused by imprecise phase modulation in traditional open-loop systems (*e*.*g*., Fresnel zone plates, FZP).

In the field of optical microscopy, aberration correction represents a central challenge for achieving high-resolution 3D dynamic observation. For techniques relying on fluorescence signal acquisition in the detection arm, the incoherent and unpolarized nature of the signal often hinders traditional methods from balancing light efficiency and aberration control. Aberration correction based on the pupil-matched remote focusing (pmRF) technique (Botcherby *et al.* 2007) hinges on precise wavefront mapping to enable aberration-free axial focusing. The pmRF technique instantaneously corrects defocus in 3D volumes for high-NA systems by accurately matching wavefronts coupled into the back pupil of the objective, ensuring high-quality, aberration-free focal control. Building on this, Chakraborty’s team (Dibaji *et al.* 2024) decomposed the detection-arm fluorescence signal into S- and P-polarized light, processed them through separate remote focusing modules, and achieved orthogonal polarization conversion using a quarter-wave plate and mirrors before recombining via a polarizing beam splitter (PBS) cube. By controlling the incident angle *θ* of the beams to minimize focal separation and integrating sub-pixel image registration, the system ensures aberration-free superposition of the two signal paths, enabling diffraction-limited axial scanning over a 70-μm range.

In open-top light-sheet fluorescence microscopy configurations, when light traverses glass interfaces (*e*.*g*., coverslips, microfluidic chips) at oblique angles, refractive index mismatches introduce aberrations. Adaptive optics (AO) techniques aim to restore imaging performance through two key steps: (1) sensing the distorted wavefront; (2) applying correction wavefronts with equal amplitude but opposite sign via adaptive elements such as deformable mirrors (DMs) or spatial light modulators (SLMs) to suppress aberrations (Johnson *et al.* 2024).

In aberration correction for dual-view light-sheet microscopy (daoSPIM), Vladimirov *et al*. proposed an AO scheme based on a deformable mirror (DM) to effectively compensate for aberrations induced by a 45°-tilted glass coverslip (Vladimirov *et al.* 2021). The design utilizes a knife-edge prism mirror to split the excitation path and combine the detection paths, enabling a single DM to correct aberrations in both orthogonal views simultaneously through symmetric optical alignment.

Hubert *et al*. developed a closed-loop AO technology using an extended-scene Shack-Hartmann wavefront sensor (ESSH), achieving efficient aberration correction via a dual-color labeling strategy and dynamic DM compensation (Hubert *et al.* 2023). The ESSH provides real-time wavefront error feedback to drive the DM, compensating for spherical aberration, astigmatism, and other aberrations. After correction, the wavefront root-mean-square (RMS) error is reduced from 256–377 nm to 34–37 nm, meeting the diffraction-limited imaging criterion (λ/15 RMS).

## AI-EMPOWERED INTELLIGENT LIGHT-SHEET MICROSCOPY IMAGING

In recent years, artificial intelligence (AI) technology has profoundly transformed the field of light-sheet fluorescence microscopy (LSFM) imaging, achieving breakthroughs through two key pathways. On one hand, during the imaging process, deep learning-based adaptive optical aberration correction techniques and computational imaging methods have significantly improved imaging quality. On the other hand, in post-imaging processing, AI empowers critical steps such as intelligent image screening, noise removal, super-resolution reconstruction, hyperspectral unmixing, and pathological analysis. This paradigm of deeply embedding AI throughout the entire imaging workflow not only overcomes the physical limitations of traditional optical systems but also drives the evolution of light-sheet microscopy toward intelligence and high precision, providing new technological tools for life science research and clinical diagnostics.

In 2023, Mani Ratnam Rai *et al*. investigated the issue of sample-induced optical aberrations in LSFM imaging (Rai *et al.* 2023). The research team employed deep learning to estimate aberrations using just two images of the same region of a tissue sample and then corrected them using a deformable mirror to enhance image quality. The study utilized a ResNet-based classification network to estimate aberrations in samples such as the pig cochlea, mouse brain, and rat brain. The team compared two network architectures – one sharing convolutional features and another independently estimating each aberration – and found that the latter provided more accurate estimations, improving image quality by 23% (shared network) and 27% (non-shared network), respectively. The method also performed well on untrained mouse brain tissue samples. This research offers an efficient approach to LSFM aberration correction, and advancing microscope imaging technology.

In 2025, the team led by Bo Dai at the University of Shanghai for Science and Technology developed a deep learning-based filter-free fluorescence microscopy (DL-F3M) technique (Dai *et al.* 2025). They constructed an optical system comprising specific LED light sources, a color camera, and a neural network processing system, trained the neural network in multiple aspects, and evaluated its performance using various cell and tissue samples. In this process, the AI-driven neural network replaced traditional optical filters, enabling filter-free fluorescence imaging and accurately extracting fluorescence signals from images with excitation light scattering. The method also improved imaging accuracy and adaptability, precisely identifying fluorescence in different samples and demonstrating robustness to new samples and variations in excitation light power. Additionally, it facilitated complex sample analysis, addressing spectral overlap in immunofluorescence detection, thereby providing strong support for biological analysis and disease diagnosis.

In the field of post-imaging processing, AI has also demonstrated multidimensional breakthroughs. In 2020, Lanxin Zhuand Chunyu Fang *et al*. proposed deep learning super-resolution light-sheet add-on microscopy (Deep-SLAM), aiming to achieve fast isotropic light-sheet fluorescence imaging of large biological samples (Zhao *et al.* 2020). The team integrated a compact add-on module into a conventional widefield microscope, transforming it into a 3D light-sheet microscope, and then combined it with a deep neural network (DNN) to enhance axial resolution. In this study, a U-Net-based CARE model was trained to reconstruct high-resolution images from low-resolution inputs, improving the system's axial resolution from 15 to 3 μm and achieving isotropic imaging at single-cell resolution. Experiments on fluorescently labeled mouse brain neurons confirmed Deep-SLAM’s advantages in resolution enhancement and image quality improvement, enabling precise neuron segmentation and cell counting. This technology equips ordinary microscopes with high-end capabilities, offering an efficient and cost-effective imaging solution for biological research.

In 2023, Anne Stockhausen *et al*. developed an Airy beam light-sheet microscope and a deep learning-based image deconvolution algorithm to address the trade-off between field of view and light-sheet thickness in traditional Gaussian beam illumination, as well as the image quality degradation caused by Airy beam side lobes (Stockhausen *et al.* 2023). The study employed a generative adversarial network (GAN)-based deconvolution algorithm, using Gaussian beam-illuminated images as training data to process Airy beam-illuminated images without requiring knowledge of the microscope’s point spread function (PSF). This improved image contrast and optimized bicubic upsampling. Experimental comparisons between Gaussian and Airy beams demonstrated that the algorithm was approximately 20 times faster than standard methods when imaging mouse brain tissue samples, providing an efficient approach for imaging and analyzing large biological specimens.

AI’s advantages in data throughput and analytical depth have also driven transformations in neuroscience and clinical medicine. In 2024, Jonathan T.C. Liu and his team addressed the limitations of traditional 2D pathological sectioning in fully characterizing tissue morphology by developing an integrated system combining light-sheet microscopy and AI (Song *et al.* 2024). The study used open-top light-sheet microscopy (OTLS) to acquire high-resolution 3D images of large tissue volumes, providing rich raw data for subsequent analysis while preserving tissue integrity for other tests. AI empowered the TriPath deep learning platform to process these images, segmenting large tissue volumes into small 2D or 3D patches, automatically extracting morphological features using a pre-trained feature encoder, and employing an attention aggregation module to weigh and combine features for accurate prediction of clinical endpoints. Experiments on simulated data and prostate cancer patient OTLS/microCT datasets showed that the system outperformed traditional 2D section analysis and clinical pathologists in risk stratification tasks, effectively reducing sampling bias and demonstrating strong generalizability in cross-modal evaluations. This work opens new avenues for the clinical application of 3D pathology.

In 2022, Joshua L. Lillvis *et al*. developed a high-throughput analysis pipeline and imaging protocol for expansion and light-sheet microscopy (ExLLSM) to rapidly reconstruct neural circuits (Lillvis *et al.* 2022). The study integrated expansion microscopy with AI, using expansion microscopy to isotropically inflate *Drosophila* brain tissue by 8-fold, reducing microscope resolution requirements and making fine structures easier to observe. Concurrently, they developed AI algorithms based on convolutional neural networks (*e*.*g*., 3D U-Net) for automated neuron segmentation and synapse classification. With this integration, ExLLSM achieved tricolor imaging of the *Drosophila* central brain at ~30 × 30 × 100 nm^3^ resolution, identifying individual electrical and chemical synapses and enabling nanoscale-resolution imaging. AI’s automated image analysis reduced manual processing time and workload, and combined with ExLLSM’s ability to image the *Drosophila* central brain in about five days while processing multiple samples in parallel, significantly increased imaging throughput. This advancement enables large-scale studies of neural circuit structure and function, facilitating a deeper understanding of neural circuit changes under different conditions.

## ADVANCEMENTS AND APPLICATIONS OF HYPERSPECTRAL LIGHT-SHEET MICROSCOPY

**Figure 2:**
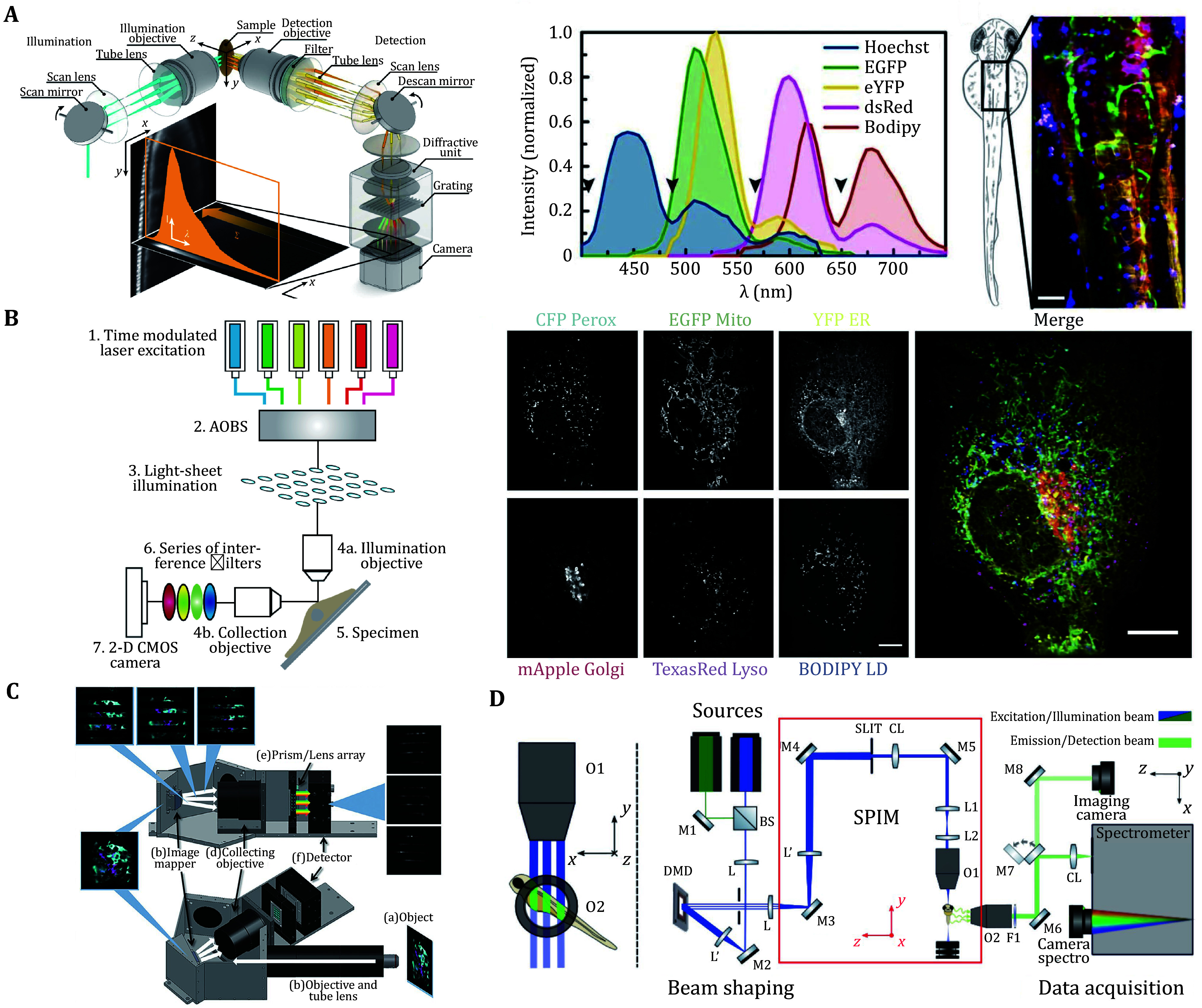
Application of light sheet microscope in hyperspectral imaging. **A** Left: DSLM with hyperspectral; Right: Five-color imaging of a zebrafish embryo (Jahr *et al.* 2015). **B** LLS with an excitation-based linear unmixing. Six different organelles in live cells (Valm *et al.* 2017). **C** IMS-iSPIM (Lavagnino *et al.* 2016). **D** Computational hyperspectral with DMD (Crombez *et al.* 2022)

Multiplexed fluorescence imaging requires spectral unmixing, traditionally achieved through optical filters. Although filter-based approaches offer simplicity, they suffer from photon loss from light outside the filter’s passband and limited spectral discrimination – particularly for fluorophores with overlapping emission profiles. To address these limitations, biological imaging increasingly integrates hyperspectral acquisition systems to leverage the full emission spectrum of fluorophores, achieving unmixing accuracy compared to filter-based methods (Fig. 2).

In 2014, Jan Huisken pioneered the integration of line-scanning light-sheet microscopy with commercial imaging spectrometers (Jahr *et al.* 2015), obtaining hyperspectral data cubes through linear unmixing. The system achieved a spectral sampling density of 0.5 nm/pixel, with descanned detection ensuring fluorescence signal stability at the spectrometer slit during dynamic scanning. Fluorescence emission was spatially dispersed via a diffraction grating, generating 3D spectral datacubes (*x*, *y*, *λ*) for each voxel. This method has been successfully applied to zebrafish and fruit fly embryos, allowing for the clear distinction of overlapping fluorescence signals and removal of autofluorescence, thus enabling detailed observation of cellular structures. However, the line-scanning modality inherently limited temporal resolution, posing challenges for real-time capture of rapid biological dynamics in living specimens.

To address these temporal constraints, David W. Piston's team pioneered a hyperspectral strategy by integrating image mapping spectrometry (IMS) (Lavagnino *et al.* 2016) with inverted selective plane illumination microscopy (iSPIM). This method reorganizes the image using a multi-faceted mirror array, strategically generating spatial voids in intermediate images. A dispersing prism then spreads the spectrum from each pixel into these voids, enabling single-snapshot acquisition of complete (*x*, *y*, *λ*) datacubes with 3 nm spectral channel spacing across 60 spectral channels and 520 nm spatial resolution. While this system represented an improvement in imaging speed, a trade-off emerged between spatial and spectral resolution, since both dimensions are captured on the same camera chip. Additionally, reliance on custom optical components and potential issues with cross-talk.

Meanwhile, the rapid evolution of spectral technologies has driven increasing demand for robust systems-level workflows adaptable to diverse applications. In 2017, an integrated multispectral imaging platform (Valm *et al.* 2017) was developed, combining lattice light-sheet (LLS) microscopy with excitation-based linear unmixing. This system employed six-laser sequential excitation under LLS illumination, enabling multiplexed analysis of dynamic inter-organelle interactions among the endoplasmic reticulum, Golgi apparatus, lysosomes, peroxisomes, mitochondria, and lipid droplets. The methodology constituted a methodological breakthrough in multi-organelle imaging, providing critical insights into the molecular mechanisms underlying organelle interactions while demonstrating LLS’s potential for complex biological interrogation.

The introduction of a computational hyperspectral imaging method in 2022 marked a paradigm shift in imaging technology. This platform (Crombez *et al.* 2022) leveraged a digital micromirror device (DMD) for spatial light modulation along the *x*-axis, coupled with a cylindrical lens that focused emitted fluorescence onto the slit of an imaging spectrometer aligned along the *y*-axis. The system achieved 2-nm spectral resolution and demonstrated live imaging capability in Hydra specimens labeled with two fluorophores. Compared with conventional scanning approaches, the technique not only enhanced temporal resolution but also eliminated the need for scanning in both directions. However, the DMD-based modulation compromised *x*-axis spatial resolution by an order of magnitude, highlighting the need for computational methods to compensate for this lateral resolution loss in future implementations.

In contrast to conventional analytical approaches, Raman spectroscopy has emerged as a powerful label-free modality capable of generating chemically resolved molecular fingerprints. While avoiding exogenous labeling, the technique nevertheless suffers from intrinsically weak signals and vulnerability to autofluorescence artifacts, constraining both detection sensitivity and quantitative accuracy. Recent advances demonstrate substantial promise in hybrid Raman-light-sheet platforms. By combining the strengths of both techniques, this approach enhances signal acquisition and spatial resolution, enabling high-quality three-dimensional imaging of complex biological and chemical samples.

To further improve Raman imaging, W. Müller *et al*. developed in 2015 a stable high-étendue interferometer (Müller *et al.* 2016), achieving extended optical travel ranges with wide angular acceptance. This innovation enabled efficient hyperspectral Raman data acquisition, and spectral unmixing was performed using non-negative matrix factorization (NMF) to enhance data analysis. The system achieved a lateral resolution of 375 nm and a spectral resolution of 4.4 cm^−1^, enabling Raman imaging of single zebrafish tissue slices in just 17 min.

However, interferometers typically sacrifice up to 50% of incident photons and require the sample to remain stationary to avoid spectral distortions. These constraints were overcome by I. Rocha-Mendoza *et al*. through the integration of an interferometric tunable filter (ITF) with a continuous-wave (CW) laser (Rocha-Mendoza *et al.* 2015), enabling high-resolution spontaneous Raman 3D imaging in light-sheet microscopy. By dynamically modulating the ITF angle, the system achieves wavelength-selective Stokes signal acquisition across spatial coordinates. This technique was successfully validated by imaging the C-H (2800−3100 cm^-^^1^) region in *C. elegans* specimens, and achieved imaging velocities surpassing conventional confocal point-scanning Raman systems by four orders of magnitude.

Traditional light-sheet microscopy relied on dual-objective configurations where high-NA objectives imposed severe spatial constraints on sample positioning. The transition to single-objective architectures resolved these operational limitations. In 2024, K. Guo *et al*. achieved the first successful integration of hyperspectral imaging with single-objective light-sheet microscopy (Guo *et al.* 2024). The system employed two orthogonal galvanometric mirrors (Galvo X and Galvo Y) to scan the focal point across the image plane. The *y*-axis acted as the spectral axis, and after grating diffraction, the camera captured the light, producing large-scale 2D datasets. With a spectral resolution of 6−11 nm, the system successfully recorded spontaneous Raman maps of injured zebrafish embryos at five-minute intervals and was able to capture the beating heart *in vivo* at video rate. This achievement marks a milestone in combining single-objective light-sheet systems with hyperspectral imaging, enabling real-time, high-resolution imaging at unprecedented speeds.

## BIOMEDICAL APPLICATIONS OF LIGHT-SHEET MICROSCOPY

**Figure 3:**
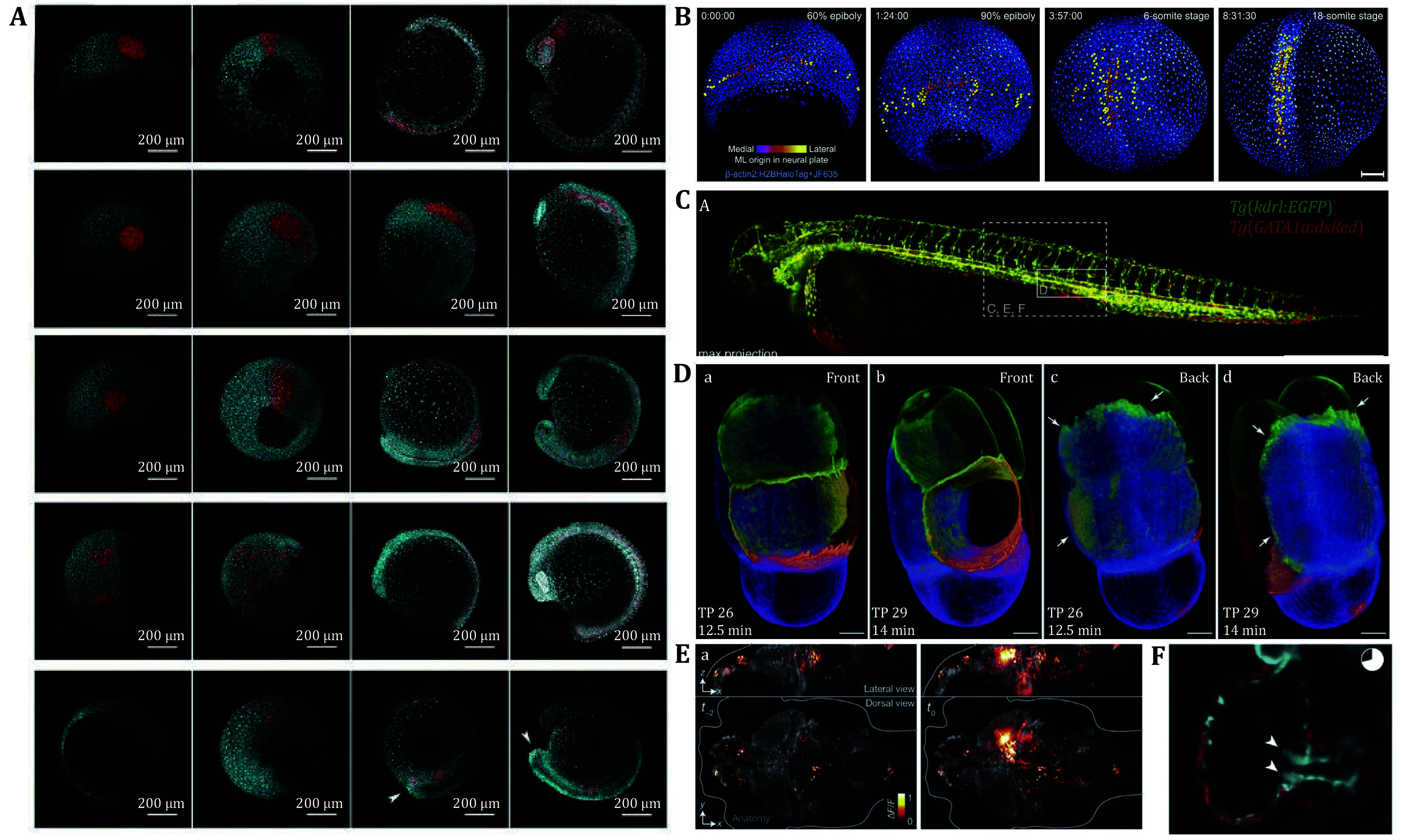
Biomedical applications of light-sheet microscopy. **A** Mechanism of tissue-level segregation between spinal cord and mesoderm populations in zebrafish embryos (Attardi *et al.* 2018). **B** MIP of zebrafish whole-embryo developmental imaging at four-time points (Wan *et al.* 2019). **C** MIP of a zebrafish whole embryo with blood vessels and red blood cells (Daetwyler *et al.* 2019). **D** Cell membranes in *C. elegans* embryos at the 4- to 6-cell stage (Fu *et al.* 2016). **E** Whole-brain imaging of neuronal activity (Ahrens *et al.* 2013). **F** The heartbeat process of zebrafish embryos (Arrenberg *et al.* 2010)

Light-sheet microscopy, characterized by its low phototoxicity, large FOV, and high-speed volumetric imaging capabilities, has emerged as a cornerstone technology for multispecies, and multiscale investigations of living systems. In both vertebrate and invertebrate models, LSM enables integrated observation of dynamic biological processes, spanning embryonic development tracking, functional neural circuit imaging, and cardiopulmonary dynamics analysis. Critically, LSM’s long-term live-imaging compatibility achieves unprecedented spatiotemporal integration of structural plasticity (*e*.*g*., tissue topology reorganization) and functional dynamics (*e*.*g*., electrophysiological propagation) within intact embryos, thereby establishing a transformative cross-scale observational paradigm for developmental biology and systems physiology (Fig. 3).

The zebrafish, owing to its embryonic transparency and rapid developmental characteristics, has become an established model for light-sheet microscopy applications. Light-sheet microscopy has revealed that neuro-mesodermal progenitors (NMps) during early zebrafish embryogenesis exhibit bipotency, differentiating into both spinal cord and mesodermal lineages. As development proceeds, these progenitors undergo differentiation at the tailbud stage, gradually committing to neural or mesodermal lineages (Attardi *et al.* 2018). Furthermore, studies have revealed the synchronization of local neural activity during spinal motor circuit assembly, offering mechanistic insights into vertebrate motor development (Wan *et al.* 2019). Additionally, three-dimensional quantification of the vascular network using this technology has uncovered spatiotemporal heterogeneity in vascular growth dynamics (Daetwyler *et al.* 2019).

In chicken embryo research, light-sheet microscopy has been achieved by integrating CUBIC tissue clearing with multispectral imaging, significantly enhancing high-resolution immunostaining visualization in late-stage embryonic brains (Gómez-Gaviro *et al.* 2017). Furthermore, this technology dynamically revealed myosin II-regulated apical contraction and cell intercalation mechanisms during primitive streak formation, providing a biomechanical model for understanding gastrulation movements in vertebrates (Rozbicki *et al.* 2015).

The mouse, a cornerstone model in mammalian research, presents technical challenges for light-sheet microscopy due to tissue opacity and long-term tracking limitations in embryonic imaging. These limitations have been addressed through optimized light-sheet systems enabling complete lineage tree reconstruction from zygote to blastocyst (Strnad *et al.* 2016), as well as multimodal imaging strategies integrating SPIM with rotational imaging optical coherence tomography (RI-OCT) to synchronously resolve embryonic morphological development and tissue biomechanical properties (Wu *et al.* 2017a).

Light-sheet microscopy has emerged as a pivotal tool in invertebrate developmental studies. In *C. elegans* and *D. melanogaster*, it resolved spatiotemporally precise protein localization and dynamics during embryogenesis (Chen *et al.* 2014). Concurrently, the RACE platform facilitated high-throughput automated analysis of morphogenetic cell behaviors across entire embryos (Stegmaier *et al.* 2016), and its synergy with biomechanical modeling delineated force-field-driven cellular rearrangements underlying gastrulation (Streichan *et al.* 2018). In *T. castaneum*, modified light-sheet systems coupled with novel sample immobilization methods achieved the first continuous whole-embryo imaging, uncovering germ layer adhesion mechanisms distinct from those in *Drosophila* (Münster *et al.* 2019; Strobl and Stelzer 2014).

Light-sheet microscopy demonstrated remarkable potential in neuroscience research, particularly in high-resolution imaging of whole-brain neural activity. By utilizing the genetically encoded calcium indicator GCaMP3, researchers identified correlations in spontaneous activity across multiple brain regions in zebrafish larvae, revealing dynamic features of functional connectivity between these regions (Panier *et al.* 2013). Subsequent advancements employing GCaMP5G enabled the near-complete recording of whole-brain neural activity in zebrafish larvae, capturing over 80% of neurons at a sampling rate of 0.8 Hz. This achievement represented the first near-exhaustive imaging of single-neuron activity in a vertebrate brain (Ahrens *et al.* 2013). In studies on invertebrates, light-sheet microscopy integrated with two-photon or one-photon excitation achieved functional imaging across the entire central nervous system (CNS) of *Drosophila* larvae at sampling rates of 2 Hz or 5 Hz, marking the first demonstration of neural activity recording at near-cellular resolution in a higher invertebrate CNS (Lemon *et al.* 2015). Collectively, these studies underscored the versatility of light-sheet microscopy as a transformative tool for cross-species investigations of neural circuits, significantly advancing the resolution of neural activity from single-cell to whole-brain scales.

In studies of cardiovascular development, targeted expression of light-sensitive proteins (halorhodopsin and channelrhodopsin) in zebrafish cardiomyocytes enabled precise optogenetic manipulation. Through this approach, researchers systematically elucidated the spatiotemporal regulation of pacemaker formation during cardiac looping morphogenesis (Arrenberg *et al.* 2010). Complementing these functional analyses, high-speed single-plane illumination microscopy (SPIM) provided real-time visualization of four-dimensional motion and structural remodeling in the zebrafish heart across entire cardiac cycles. This technique resolved previously uncharacterized relationships between cardiac anatomy and hemodynamic function (Mickoleit *et al.* 2014; Taylor *et al.* 2019). Beyond vertebrate models, light-sheet microscopy achieved unprecedented temporal resolution in developmental biology by documenting the complete morphogenetic trajectory of the *Drosophila* embryonic tracheal system. Through continuous 95-min imaging of all tracheal branches, the study delineated the real-time dynamics of branch elongation, bifurcation, and lumen formation, establishing a quantitative framework for analyzing tubular organogenesis (Aiswarya *et al.* 2024). These paradigm-shifting applications collectively highlight the capacity of light-sheet microscopy to integrate functional perturbation, structural dynamics, and developmental chronology in complex living systems.

## SUMMARY AND PERSPECTIVES

Recent advancements in single-objective light-sheet microscopy have significantly improved system compatibility. However, the introduction of oblique plane imaging technology has introduced new challenges, including numerical aperture (NA) loss, the complexity of aligning tertiary objectives with focal planes, and the limitations of remote focusing techniques on imaging magnification. These issues hinder the system's ability to perform in situ multi-scale observations in scenarios requiring variable magnification. In recent years, novel optical designs, such as the TIM system, have addressed these challenges by eliminating the oblique plane correction module, enabling seamless 4X-100X magnification switching. This innovation provides a flexible solution for cross-scale dynamic studies.

In the realm of hyperspectral integration, existing technologies face a fundamental conflict between the two-dimensional excitation characteristics of light sheets and the three-dimensional data requirements of spectral detection. This conflict often forces a trade-off between spatial resolution (*e*.*g*., using DMD-based spectral splitting) and temporal resolution (*e*.*g*., line-scanning spectral splitting), making it difficult to meet the demands of high-speed, multi-color imaging in live specimens. Moving forward, there is an urgent need to develop new light-sheet-spectral coupling architectures. By optimizing optical pathways and integrating computational imaging, these systems could maintain subcellular resolution and millisecond-level temporal precision while enabling simultaneous multi-spectral channel acquisition. Such advancements would propel the application of five-dimensional imaging (*x*, *y*, *z*, *t*, *λ*) in single-cell interactions, organoid development, live embryo dynamics, and centimeter-scale tissue clearing. Ultimately, these technologies aim to establish a multi-modal, high-throughput, and intelligent panoramic imaging paradigm, providing life sciences with a comprehensive research tool that bridges molecular networks and systems biology.

Looking at recent progress, AI is reshaping the technological ecosystem of light-sheet microscopy. At the hardware level, strategies such as aberration correction and filter replacement simplify system architecture and significantly reduce costs. In data processing, super-resolution reconstruction and deconvolution optimization enhance imaging precision, pushing axial resolution into the micrometer and even nanometer range. In applications, automated segmentation and 3D feature extraction multiply imaging throughput and extend its reach to clinical diagnostics. These breakthroughs not only demonstrate AI’s core advantages in improving imaging speed, precision, and adaptability but also reveal its future potential – such as closed-loop intelligent systems combining real-time aberration correction with adaptive illumination, or leveraging transfer learning to adapt lab models for portable devices, enabling rapid bedside diagnostics. As algorithms and optical design deepen their integration, AI is poised to further break the performance boundaries of light-sheet microscopy, providing even more powerful tools for life science and medical research.
